# Transferability of Dual-Task Coordination Skills after Practice with Changing Component Tasks

**DOI:** 10.3389/fpsyg.2017.00956

**Published:** 2017-06-13

**Authors:** Torsten Schubert, Roman Liepelt, Sebastian Kübler, Tilo Strobach

**Affiliations:** ^1^Department of Psychology, Martin-Luther University Halle-WittenbergHalle, Germany; ^2^Institute of Psychology, German Sport University CologneCologne, Germany; ^3^Department of Psychology, Medical School HamburgHamburg, Germany

**Keywords:** dual tasks, practice, executive functions, task coordination skills, transfer

## Abstract

Recent research has demonstrated that dual-task performance with two simultaneously presented tasks can be substantially improved as a result of practice. Among other mechanisms, theories of dual-task practice-relate this improvement to the acquisition of task coordination skills. These skills are assumed (1) to result from dual-task practice, but not from single-task practice, and (2) to be independent from the specific stimulus and response mappings during the practice situation and, therefore, transferable to new dual task situations. The present study is the first that provides an elaborated test of these assumptions in a context with well-controllable practice and transfer situations. To this end, we compared the effects of dual-task and single-task practice with a visual and an auditory sensory-motor component task on the dual-task performance in a subsequent transfer session. Importantly, stimulus and stimulus-response mapping conditions in the two component tasks changed repeatedly during practice sessions, which prevents that automatized stimulus-response associations may be transferred from practice to transfer. Dual-task performance was found to be improved after practice with the dual tasks in contrast to the single-task practice. These findings are consistent with the assumption that coordination skills had been acquired, which can be transferred to other dual-task situations independently on the specific stimulus and response mapping conditions of the practiced component tasks.

## Introduction

Performing two component tasks simultaneously at the same time (i.e., dual tasks) can be extremely difficult but this difficulty is often reduced after practice. For example, during the first lessons of driving school students find it challenging to coordinate the large number of different activities and components of car driving (e.g., changing gear, lane change, navigation, etc.). At the end of the lessons students are, however, able to coordinate these activities enabling them to drive safely in road traffic. In other words, an improved coordination of multiple task requirements may result from ongoing practice and may lead to improved performance in dual-task-like situations of car driving at the end of practice. However, while this example illustrates a plausible every day situation of practice-related improvement in dual-task coordination, findings in the literature are not conclusive concerning empirical evidence for the acquisition of task coordination skills to explain dual-task improvement.

The present study tackles this issue, assessing whether task coordination skills are acquired with dual-task practice and whether this represents a mechanism underlying improved dual-task performance, which may explain practice-related reduction of dual-task costs. The improvement of task coordination skills is typically associated with an optimized control of two simultaneous task processing streams in dual-task situations (Hirst et al., 1980). From a learning perspective, it is important to know whether there is evidence for such skill acquisition (Anderson, 1988; Taatgen, 2013) in the context of complex situations (i.e., situations with two simultaneous tasks). Such evidence would be important for learning accounts assuming that task coordination skills can be acquired during dual-task training and that these skills are assumed to affect the practice-related improvement of dual-task performance in addition to task automatization (Schneider and Shiffrin, 1977; Logan, 1988) or learning of the component tasks during dual-task practice (Ahissar et al., 2001; Ruthruff et al., 2006; Maquestiaux et al., 2008; Strobach et al., 2013; Strobach and Schubert, 2017).

### Prior Tests of Task Coordination Skills

According to theoretical considerations, task coordination skills are acquired and improved under dual-task practice conditions, but not when practicing each task in isolation under single-task practice conditions (Hirst et al., 1980; Kramer et al., 1995; Strobach et al., 2014). That is, these skills evolve from practicing two tasks simultaneously, rather than being attributable to learning the component tasks (Damos and Wickens, 1980; Oberauer and Kliegl, 2004; Silsupadol et al., 2009). Furthermore, once acquired, improved task coordination skills should at least be partially independent of the specific properties of the component tasks presented during dual-task practice. Consequently, these skills should be (at least to some extent) transferable across different dual-task situations (Kramer et al., 1995; Bherer et al., 2006; Liepelt et al., 2011).

Several studies have provided preliminary evidence, which supports the assumption that under certain conditions task coordination skills can indeed be acquired during long lasting training. As an example, studies testing the consequences of experience with dual tasks in comparison with the consequences of single task training were conducted in persons having extensive experience in playing a particular type of video games, known as action video games and its common subgenre ego-shooters. These games typically require the fast performance of several actions such as follow and maintain aims of the game, fight enemies, locate supplies, etc. The actions are performed under extreme temporal processing demands either at the same time or within close temporal proximity during the games. In contrast to persons without game experience, action video gamers show an optimized ability to perform and coordinate two simultaneous tasks in a dual-task paradigm of the Psychological Refractory Period (PRP) type (Pashler, 1994; Schubert, 1999) including well-controllable sensorimotor tasks (e.g., Strobach et al., 2012c; Chiappe et al., 2013).

Further evidence suggesting that task coordination skill may differ between persons with different degree of multi-tasking experience, stems from studies comparing the dual-task performance of highly skilled simultaneous interpreters and control participants. Simultaneous interpreting is an activity that is highly complex and requires the performance of multiple simultaneous tasks. Among others, these tasks include the analysis and understanding of the discourse in a first language, reformulating linguistic material, language production in a second language, and storing of intermediate processing steps. In two studies, we explored whether persons with experience in simultaneous interpreting possess superior skills in coordination of multiple tasks and whether they are able to transfer these skills to PRP dual tasks (Strobach et al., 2015a; Becker et al., 2016). In fact, we found faster dual-task reaction times (RTs) in persons with experience in simultaneous interpretation in contrast to control participants without such experience. Thus, action video gamers and simultaneous interpreters seem to possess superior skills to coordinate multiple tasks in lab-based dual-task situations. However, both cases, action video gaming as well as simultaneous interpreting, are no well-controllable practice situations and, therefore, do not allow for a systematic analysis of the specific underlying mechanisms and practice components as well as of the amount of practice enabling the acquisition of task coordination skills. For instance, in the context of video games, the number and frequency of situations with the presentation of simultaneous tasks is uncontrolled, so are the types of tasks combined. Further, it might be the sheer complexity of the situations (i.e., the combination of multiple tasks in action video games or during simultaneous interpreting without temporal overlap of these tasks), but not the experience of simultaneous tasks *per se*, that had led to the acquisition of task coordination skills (Schneider et al., 2002).

Recently, several studies proposed a well-controllable situation of dual-task practice, which is promising to investigate the characteristics of task coordination skill acquisition (Liepelt et al., 2011; Strobach et al., 2012d). In that situation, two groups of participants experience different types of practice with two sensorimotor component tasks, a visual-manual (i.e., the visual task) and an auditory-verbal tasks (i.e., the auditory task). In the visual task, there were spatially compatible mappings of circle positions on the screen to manual finger responses. In the auditory task, different tone pitches (low, medium, high) had a compatible mapping on number words (“ONE,” “TWO,” “THREE”). While *hybrid practice* included single-task and dual-task trials (see also Schumacher et al., 2001; Strobach et al., 2015b), single-task practice included single-task trials alone. After extended practice the authors compared the performance of the two different practice groups, i.e., the single-task and the hybrid practice groups, in a dual-task transfer situation in which the auditory and the visual task were processed simultaneously. In fact, after hybrid practice dual-task performance was better than after single-task practice.

**FIGURE 1:**
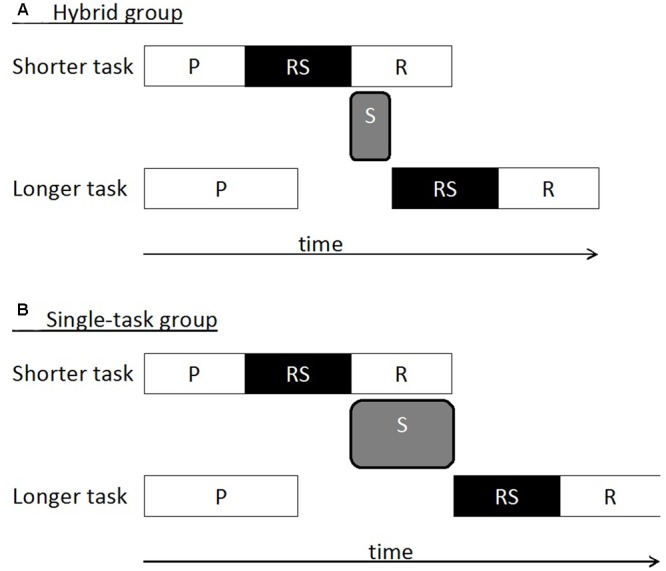
Dual-task processing architecture after hybrid practice (**A**: Hybrid group) and single-task practice (**B**: Single-task group). According to bottleneck models, central response-selection (RS) stages are processed sequentially even with task practice (Maquestiaux et al., 2004; Ruthruff et al., 2006) while perception (P) and response (R) stages are processed in parallel. A potential switching stage (S) after the response-selection (RS) stage in the shorter task and before the RS stage in the longer task is shortened after hybrid in contrast to single-task practice. The latter phenomenon is a promising candidate to explain reduced dual-task costs after having experience with dual tasks (i.e., hybrid practice). Note that the latencies of the individual processing stages (i.e., P, RS, S, R) in the figure are schematic illustrations and may not represent actual stage latencies.

Importantly, the studies could demonstrate an improvement after hybrid practice, which was exclusively realized by reduced dual-task RTs in the auditory task while there was no evidence for a hybrid practice advantage in the visual task. This is important because the auditory task was processed more slowly as compared to the visual task (see also Schumacher et al., 2001; Tombu and Jolicoeur, 2004; Hartley et al., 2011; Strobach et al., 2012a,b). The authors proposed a model, which is illustrated in **Figures 1A,B**, to explain the observation of the hybrid-practice advantage in the longer auditory task but not the shorter visual task. As can be seen in the related figures, the model is based on the well-known assumption that sensorimotor tasks can be separated into an initial perception stage, a central response-selection stage and a final motor stage. According to the prominent central bottleneck model, the perception and motor stages in two tasks run in parallel while the central response-selection stages of such tasks are capacity-limited and represent bottleneck processes. This capacity limitation requires the serial processing of these stages and their coordination. **Figure 1A** illustrates the assumption that dual-task processing can be considered as the sequence of a capacity limitation in the faster visual task (e.g., at a central response-selection stage) followed by a switching operation between the response-selection stages, and the capacity limitation in the slower auditory task (Lien et al., 2003; Band and van Nes, 2006; Schubert, 2008). The switching operation is theorized as activating and/or instantiating the rules that map the stimuli of the longer task onto responses (Maquestiaux et al., 2004). It may be that these rules must be moved back into working memory or that the rules remaining in working memory throughout the task must be reestablished during ongoing processing of task 1 and task 2. After hybrid practice (**Figure 1A**) in contrast to single-task practice (**Figure 1B**), activation/instantiation processes are highly efficient due to task coordination skills, leading to a more efficient and therefore faster switching operation; in other words, participants have learned to load task information faster or more efficiently into the working memory as a result of hybrid practice. Therefore, improved dual-task performance after hybrid practice may occur in the longer auditory task, because the shortened switching operation is located between the response-selection stages in the faster visual task and the slower auditory task (Strobach et al., 2014, for a more detailed discussion).

Essentially, the conclusion of a shortened switching operation as an explanation for the findings of Liepelt et al. (2011) and Strobach et al. (2012d) can be distinguished from other explanations focusing on task automatization and/or stage shortening within the component tasks (Ruthruff et al., 2006; Strobach et al., 2013). This is so because, participants of the hybrid and single-task practice groups received the identical number of stimulus contacts and, thus, of experience with each component task during practice. In addition, the performance in the single visual task and single auditory task was similar across groups after single and hybrid practice. This makes it implausible from a methodological perspective as well as from a results perspective to assume that differences in single-task performance between the training groups can explain the findings of Liepelt et al. (2011) and Strobach et al. (2012d).

Nevertheless, several methodological aspects of the findings of Liepelt et al. (2011) and of Strobach et al. (2012d) require further elucidation of the assumption that hybrid practice with the current type of component tasks can lead to the acquisition of transferable task coordination skills. This is so because in these studies the advantage after hybrid practice in the auditory task was evident when the authors analyzed the dual-task performance in a specific transfer situation, which was presented to the participants after the final training session. More specifically, in the studies of Liepelt et al. (2011) and Strobach et al. (2012d) the dual-task situation in the transfer session was either completely identical to the practiced situation, or it consisted of at least one unchanged but practiced component task while the other component task had changed from practice to transfer; note that in different experiments either the visual or the auditory task remained unchanged from practice to transfer.

The authors interpreted these findings (i.e., transfer in conditions with one task changed while the other task remained unchanged) as evidence for the assumption that task coordination skills are not tied to specific characteristics of the practiced component tasks (Liepelt et al., 2011; Strobach et al., 2012d). While this assumption was based on the fact that transfer was found either in a situation in which the auditory component task had changed and in a different situation in which the visual task had changed from training to transfer, one cannot completely be sure that the observed findings indeed showed the existence of task coordination skills that are unspecific to the two component tasks. Thus, it might be the case that the acquired task coordination skills are tied to either of the two component tasks in a dual-task situation and only one constant task after practice might be sufficient for a successful application of acquired coordination skills during transfer. Note that a situation with at least one unchanged task remaining constant between training and transfer might allow for precisely predicting the time durations when the processes of a component task need to be started in a dual-task situation and/or are expected to be finished; this might produce a benefit for the transfer of coordination skills after practice. We know from investigations on time duration production skills, that transfer from practiced to un-practiced time durations is impaired when a secondary task presented during practice was removed during the transfer test (Healy et al., 2005).

In sum, prior studies lack convincing empirical evidence for the existence of task coordination skills, which can be transferred in task contexts with two changing component tasks during training of well-controllable task and training situations. The aim of the present study is to fill this gap in the dual-task practice literature.

Findings of earlier studies are tempting to assume that an increase in the variability of the practiced learning examples in alternative practice situations may enforce skill transferability after practice (Schmidt and Bjork, 1992; but see Logan, 1990). For instance, the transferability of duration production skills to un-practiced durations were increased after variable practice with mixed durations in contrast to blocked sequences of trials all of the same duration in the study of Schneider et al. (1995). Therefore, in the current study, the specific stimulus and stimulus-response rules of both, i.e., of the visual and of the auditory tasks (Liepelt et al., 2011) were changed between every second practice sessions to increase the variability of the learning examples during practice; this should enforce the need to train general task coordination skills but not specific task automatization.

One group of participants trained the two tasks in 15 hybrid dual-task training sessions and these participants were tested in a final transfer Session 16 at the end of practice. A further group of single-task learners trained the two tasks in single task regimen and were also tested for dual-task performance in this transfer session; these participants also experienced the two session-wise changes of the stimuli and the stimulus-response rules of the component tasks, i.e., the specific character of the visual and auditory tasks was changed between practice sessions equivalently to the changes in the hybrid group. Importantly, the single-task group, had experience only with single-task trials during the 15 practice sessions before the final transfer Session 16. Based on the assumption that an increase in variability increases the chances for transfer of task coordination skills, we predict improved dual-task performance in transfer Session 16 after variable hybrid practice than after single-task practice.

## Materials and Methods

### Participants

Participants were randomly assigned to one of the two experimental groups: the hybrid group and the single-task group. In line with previous dual-task learning studies (Liepelt, 2011; Strobach et al., 2012d), we included eight participants (six female) with a mean age of *M* = 24.5 years (*SD* = 3.5 years) and an age range from 19 to 29 years in the hybrid group. Eight participants (five females) were included in the single-task group with a mean age of *M* = 23.8 years (*SD* = 3.2 years, age range from 19 to 28 years). According to the experience from earlier training studies (Liepelt et al., 2011; Strobach et al., 2012d), the administration of frequently changing stimulus-response rules should require a large number of training sessions in order to get reliable training-related improvements in task performance. A group size of eight participants in each group allows bringing together the requirements for an increased number of training sessions with the requirements of feasible experimental economics (Schubert and Strobach, 2012). Furthermore, an additional analysis with G^∗^Power (Faul et al., 2007) using values of previous training studies (Liepelt et al., 2011; Strobach et al., 2012d) has shown that a group size of eight participants per group will provide sufficient power (>0.9) with an alpha set at 0.05. The two groups performed altogether 16 sessions, which represents a volume of 256 h of experimentation. All participants of these groups were included into the final data set. Participants were contacted through flyers and electronic mails. All participants had normal or corrected to normal vision and were not informed of the purpose of the experiment. They were paid for participation at a rate of 8€ per session plus performance-based bonuses.

### Apparatus

Visual stimuli were presented on a 17-inch color monitor and auditory stimuli were presented via headphones, which were connected to a Pentium I IBM-compatible PC. The RT for manual responses was recorded with a button box and the RT of verbal responses was recorded via a voice key connected to the experimental computer. The experimenter typed the actual response on a computer keyboard so that accuracy could be assessed in the analysis. The experiment was controlled by the software package ERTS (*Experimental Runtime System*; Beringer, 2000).

### Stimuli and Component Tasks

**FIGURE 2:**
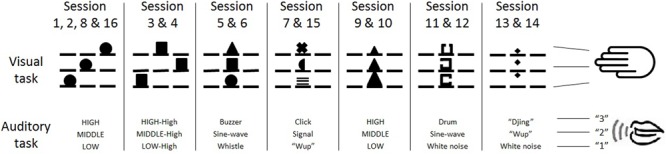
Illustration of the stimulus-response mapping characteristics of the visual and auditory tasks in the hybrid and single-task group across Sessions 1–16. See main text for further details on these characteristics.

During Sessions 1–16, participants conducted different versions of visual and auditory sensorimotor tasks. All tasks and versions were three-choice tasks and included mappings between 3 stimuli and 3 responses. In the visual task versions, all visual stimuli were white and participants responded with their index, middle, and ring finger of their right hand in accordance to the following lists of stimuli as illustrated in **Figure 2**: circles appearing at the left, central, or right screen position (Sessions 1, 2, 8, and 16), squares appearing at the left, right, or central positions (Sessions 3 and 4), a circle, square, and triangle appearing at the central position (Sessions 5 and 6), a line pattern, semicircle, and cross appearing at the central position (Sessions 7 and 15), triangles of large, medium, and small size appearing at the central position (Sessions 9 and 10), a right-, left-, or top-oriented opening in a square appearing at the central position (Sessions 11 and 12), and a diamond appearing at vertical top, central, and bottom positions (Sessions 13 and 14). In the auditory task versions, participants responded with the verbal number words “ONE,” “TWO,” and “THREE” in accordance to the following stimuli (**Figure 2**): a low, middle, and high sine-wave tone (Sessions 1, 2, 8, 9, 10, and 16), a low high-pitched, middle high-pitched, and high high-pitched sine-wave tone (Sessions 3 and 4), a whistling sound, middle sine-wave tone, and buzzer tone (Sessions 5 and 6), a “wup”-like sound, signal sound, and clicking noise (Sessions 7 and 15), high white noise, middle sine-wave tone, and low drum sound (Sessions 11 and 12), white noise, middle “wup”-like sound, and high “djing”-like sound (Sessions 13 and 14). We selected these sets of stimulus and response mappings across the visual and auditory tasks and the experimental sessions, because these mappings significantly differ from each other and thus increased variability on the one hand (e.g., stimulus-response mapping rules were compatible, incompatible, and arbitrary). On the other hand, we assumed that participants were able to perform these mappings after only a short introduction.

In visual single-task trials, three white dashes served as placeholders for the possible positions of the visual stimuli. They appeared as a warning signal 500 ms before the visual stimulus was presented. The stimulus remained visible until the participant responded or a 2,000 ms response interval had expired. After correct responses, RTs were presented for 1,500 ms on the screen. Following incorrect responses, the word “ERROR” (German: “FEHLER”) appeared. A blank interval of 700 ms preceded the beginning of the next trial. An auditory single-task trial started with the presentation of three dashes on the computer screen. After an interval of 500 ms, the tones were presented. The trial was completed when the participant responded verbally or a 2,000 ms response interval had expired. To acquire an accurate measurement of verbal responses, the experimenter typed the actual response on a computer keyboard so that accuracy could be assessed in the analysis. After verbal responses, RTs were presented for 1,500 ms on the screen. Following omitted verbal responses, the word “ERROR” (German: “FEHLER”) appeared. A blank interval of 700 ms preceded the beginning of the next trial. Dual-task trials included the visual and the auditory task. These trials were identical to single-task trials with the exception that a visual and an auditory stimulus were presented simultaneously (SOA = 0 ms). As in previous studies on a similar dual-task procedure, participants were not told to respond in any particular order and they should give equal priority to the two tasks. Instructions were designed to encourage participants to perform the tasks as quickly and accurately as possible in all trials and blocks.

### Design and Procedure

#### Hybrid Group

This group performed hybrid practice in Sessions 1–16. Each session lasted <60 min and these sessions were conducted on consecutive days (except weekends). During hybrid practice, there were single-task trials and dual-task trials. Single tasks of the visual or the auditory task were included into single-task blocks of 45 trials. In contrast, 18 dual-task trials were included into mixed blocks combined with 30 mixed single-task trials, 15 of the visual task and 15 of the auditory task. These mixed single-task trials helped to ensure that participants were equally prepared for both tasks in mixed blocks; alternatively, they could prepare for only one task that is executed first in dual-task trials. Participants were instructed to respond to both stimuli as quickly and accurately as possible during all blocks. Response order was free.

In Session 1, participants of the hybrid group performed six visual and six auditory single-task blocks that were presented in alternating order (**Table 1**). Half of the participants started with a visual single-task block and the other half with an auditory single-task block. Session 2 included six single-task blocks (three visual and three auditory task blocks) and eight mixed blocks. After two initial single-task blocks (one visual and one auditory single-task block), sequences of two mixed blocks and one single-task block followed; the type of single-task blocks was alternated. The order of blocks (first visual or auditory task block) was counterbalanced across participants. The design in Sessions 3–16 was identical to that in Session 2 but these sessions included two additional mixed blocks at the end.

**Table 1:** Illustration of the training regime across 16 sessions in the hybrid group/single-task group.

Session				
Block	1	2	3–15	16
1	s-short/s-short	s-short/s-short	s-short/s-short	s-short/s-short
2	s-short/s-short	s-short/s-short	s-short/s-short	s-short/s-short
3	s-short/s-short	mix/mix	mix/s-long	mix/mix
4	s-short/s-short	mix/mix	mix/s-long	mix/mix
5	s-short/s-short	s-short/s-short	s-short/s-short	s-short/s-short
6	s-short/s-short	mix/s-long	mix/s-long	mix/mix
7	s-short/s-short	mix/s-long	mix/s-long	mix/mix
8	s-short/s-short	s-short/s-short	s-short/s-short	s-short/s-short
9	s-short/s-short	mix/s-long	mix/s-long	mix/mix
10	s-short/s-short	mix/s-long	mix/s-long	mix/mix
11	s-short/s-short	s-short/s-short	s-short/s-short	s-short/s-short
12	s-short/s-short	mix/s-long	mix/s-long	mix/mix
13		mix/s-long	mix/s-long	mix/mix
14		s-short/s-short	s-short/s-short	s-short/s-short
15			mix/s-long	mix/mix
16			mix/s-long	mix/mix

*s-short and *s-long* indicate short single-task blocks (45 trials) and long single-task blocks (66 trials), respectively, of either the visual or the auditory task. *mix* illustrates mixed blocks (including 15 visual single-task trials, 15 auditory single-task trials, and 18 dual-task trials).*

#### Single-Task Group

The experimental procedure in the single-task group was similar to the hybrid group with the exception that this group of participants performed single tasks (almost) exclusively in Sessions 1–15 (**Table 1**). To keep the number of stimulus contacts between dual-task conditions (in the hybrid group) and single-task conditions constant, one dual-task trial was replaced by one single-task trial of each task. Consequently, we had single-task blocks with 45 trials (short blocks) or 66 trials (long blocks). Session 1 was identical to the hybrid group. Session 2 included 12 single-task blocks (six visual and six auditory task blocks). Importantly, this session also included two mixed blocks. These mixed blocks were included to analyze initial dual-task performance in the single-task practice group before practice and to match this performance between practice groups. In Session 2, these two initial mixed blocks were introduced after two short single-task blocks. Then, sequences of one short and two long single-task blocks followed. In Sessions 3–15, we presented 16 single-task blocks (eight visual and eight auditory task blocks). After two initial short single-task blocks, sequences of two long single-task blocks and one short single-task block followed. In Sessions 2–15, blocks with the visual and auditory task were alternated and the first type of block (either visual or auditory task) was counterbalanced between subjects. The following Session 16 was identical to this session in the hybrid group.

## Results

### Statistical Analyses

We excluded all trials in which responses were omitted or incorrect (7.0%) prior to statistical RT analyses. The alpha level for significant effects and interaction was set to *p* = 0.05. Effects sizes were illustrated with partial η^2^ for significant main effects and interactions.

To obtain a strong and reliable parameter for dual-task performance, we assessed dual-task performance in dual-task trials and single tasks of single-task blocks: dual-task costs = Performance_dual-tasktrials_ – Performance_single-task trialsofsingle-taskblocks_ (Tombu and Jolicoeur, 2004; Strobach et al., 2015c). This parameter of dual-task costs is particularly essential when investigating task coordination skills (Liepelt et al., 2011; Strobach et al., 2012d). It combines trials that are by definition not related to each other (i.e., pure single-task trials and dual-task trials) and is therefore most informative to investigate task coordination skills. We thus excluded mixed single-task trials from the test on skill acquisition because processing associated with this type of trials is less specified. That is, participants will also be partially prepared for a task type that did not occur and this omission of an expected stimulus and task may have thrown off or surprised subjects (Tombu and Jolicoeur, 2004).

We focused on Sessions 2–15 to analyze hybrid practice performance; note that there were no dual-task trials included in Session 1. And we focused on Sessions 1–15 when comparing the single-task performance during hybrid and single-task practice. When testing the acquisition and transfer of task coordination skills, we analyzed single-task and dual-task performance during pre-test and post-test. For the pre-test, we analyzed the dual-task performance by comparing the data in the first two single-task blocks with that of the dual-task trials in the two following mixed blocks in Session 2 in both, the hybrid and the single-task group; note that also the single-task group performed two mixed blocks after two single-task blocks in the beginning of this session. The data of Session 16 (in which both the single-task and hybrid groups performed single and dual tasks) served as the post-test measure for the performance at the end of practice.

### Hybrid and Single-Task Practice Performance

**FIGURE 3:**
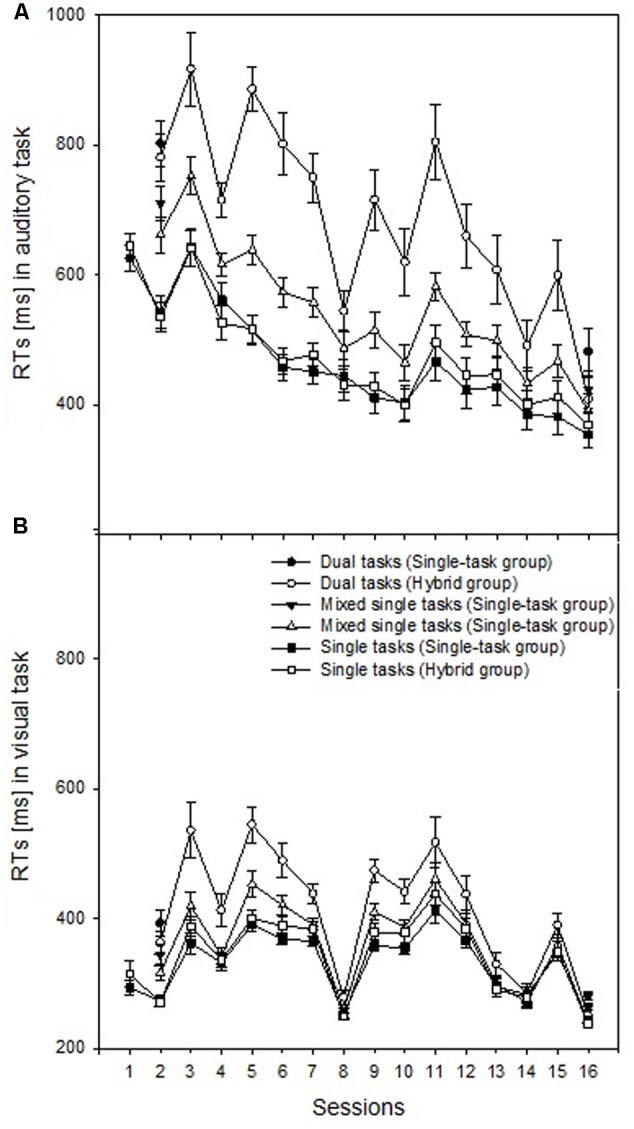
Reaction times (RTs) in millisecond (ms) of single-task trials in single-task blocks (Single tasks), single-task trials in mixed blocks (mixed single tasks), and dual-task trials (Dual tasks) for the single-task practice group (Single-task group) and the hybrid practice group (Hybrid group). **(A)** Auditory-task data; **(B)** Visual-task data.

The RT and error data of the practice sessions are illustrated in **Figure 3** and **Table 2**, respectively. To analyze dual-task performance in the hybrid group across practice, we included the within-subject factors Session (Sessions 2–15) and Trial type (dual tasks vs. single tasks) in mixed measures ANOVAs. The single-task and dual-task RTs varied as an effect of changes in the stimulus-response mapping characteristics and hybrid practice in the auditory task, as indicated by main effects of Session *F*(13,91) = 28.530, *p* < 0.001, $\eta_{p}^{2}$ = 0.80, and Trial type, *F*(1,7) = 62.459, *p* < 0.001, $\eta_{p}^{2}$ = 0.90, as well as the interaction, *F*(13,91) = 16.801, *p* < 0.001, $\eta_{p}^{2}$ = 0.71 (**Figure 3A**). The visual-task RTs showed a similar pattern with main effects of Session, *F*(13,91) = 31.710, *p* < 0.001, $\eta_{p}^{2}$ = 0.82, and Trial type, *F*(1,7) = 55.972, *p* < 0.001, $\eta_{p}^{2}$ = 0.89, as well as the interaction of Session and Trial type, *F*(13,91) = 5.028, *p* < 0.001, $\eta_{p}^{2}$ = 0.42 (**Figure 3B**). Similar to the auditory-task RTs, this task’s error rates also varied with hybrid practice and changes in the stimulus-response mapping characteristics, as indicated by main effects of Session, *F*(13,91) = 5.502, *p* < 0.001, $\eta_{p}^{2}$ = 0.44, and Trial type, *F*(1,7) = 97.811, *p* < 0.001, $\eta_{p}^{2}$ = 0.93, as well as their interaction, *F*(13,91) = 6.823, *p* < 0.001, $\eta_{p}^{2}$ = 0.49. The visual-task error rates showed a similar pattern with main effects of Session, *F*(13,91) = 5.256, *p* < 0.001, $\eta_{p}^{2}$ = 0.43, and Trial type, *F*(1,7) = 11.767, *p* < 0.001, $\eta_{p}^{2}$ = 0.63, as well as the interaction, *F*(13,91) = 4.873, *p* < 0.001, $\eta_{p}^{2}$ = 0.41.

**Table 2:** Error rates (in percent) across the auditory and visual tasks, the single-task, mixed single-task, and dual-task conditions, from Session 1 to Session 16 in the hybrid and single-task groups.

	Hybrid group						Single-task group					
	Auditory task			Visual task			Auditory task			Visual task		
Session	Single tasks	Mixed single tasks	Dual tasks	Single tasks	Mixed single tasks	Dual tasks	Single tasks	Mixed single tasks	Dual tasks	Single tasks	Mixed single tasks	Dual tasks
1	7.0			0.6			6.1			1.5		
2	4.4	5.6	11.6	2.0	0.6	4.3	4.3			1.8		
3	7.1	8.6	23.1	3.1	4.0	12.4	7.0			4.1		
4	4.7	9.6	13.9	3.4	2.6	4.5	6.1			3.2		
5	3.6	4.7	21.9	5.7	5.5	12.8	3.2			4.6		
6	3.4	3.1	12.4	4.0	3.2	8.1	3.9			4.1		
7	3.9	3.8	6.5	4.3	4.3	6.0	4.4			4.5		
8	5.2	9.0	9.4	3.4	1.3	1.9	5.9			3.5		
9	5.6	5.8	17.1	7.8	7.6	8.5	5.8			6.2		
10	4.2	4.6	14.4	4.0	7.0	7.7	5.3			4.1		
11	6.6	5.6	20.5	7.9	6.5	12.3	6.6			5.2		
12	3.6	5.1	16.3	7.4	5.9	8.1	5.4			4.4		
13	6.4	7.2	13.1	5.5	4.6	5.6	8.1			4.0		
14	4.8	7.6	11.3	5.3	4.7	3.9	6.7			3.8		
15	5.2	6.3	4.4	6.8	7.7	7.2	5.4			4.6		
16	7.3	11.8	12.1	4.9	2.0	3.5	8.3	12.3	15.2	2.9	2.0	3.4

To demonstrate similar levels of component-task processing skills during practice and transfer, we analyzed the single-task trials of single-task blocks in mixed measures ANOVA including the within-subjects factor Session (Sessions 1–15) and the between-subjects factor Group (hybrid group vs. single-task group); note that potential effects and interactions with Group were mainly relevant in these analyses. The auditory single-task RTs (**Figure 3A**) showed no main effect or interaction with Group, *F*s < 0.913, *p*s > 0.545; Session demonstrated variable RTs across practice and changing task characteristics, *F*(1,14) = 66.456, *p* < 0.001, $\eta_{p}^{2}$ = 0.83. The error data in this task showed no main effect of and interaction with Group, *F*s < 0.910, *p*s > 0.549; Session was significant, *F*(1,14) = 9.381, *p* < 0.001, $\eta_{p}^{2}$ = 0.40 (**Table 2**). The visual single-task RTs (**Figure 3B**) produced no main effect of and interaction with Group, *F*s < 0.910, *p*s > 0.549; Session demonstrated variable RTs across practice and changing task characteristics, *F*(1,14) = 69.854, *p* < 0.001, $\eta_{p}^{2}$ = 0.83. The main effect of and interaction with Group were also not evident in the error analysis of the visual task, *F*s < 1.219, *p*s > 0.29; Session was significant, *F*(1,14) = 8.976, *p* < 0.001, $\eta_{p}^{2}$ = 0.38 (**Table 2**). Thus, component-task processing skills did not statistically differ between both groups across practice.

### Transfer Test on Task Coordination Skills

In this section, we compare the dual-task costs at the beginning of practice (i.e., pre-test: first two single-task blocks and dual-task trials of the first two mixed blocks in Session 2) and at the end of practice (i.e., post-test: single-task blocks and dual-task trials of the mixed blocks in Session 16) in the hybrid and the single-task group. Reduced dual-task costs and improved dual-task performance in the hybrid group, compared to the single-task group, during post-test would indicate the acquisition and transfer of improved task coordination skills if controlled for possible performance differences in the pre-test. In particular, as illustrated in **Figure 1**, the improved dual-task performance in the hybrid group is expected in the auditory task, because the anticipated speed-up switching operation is located between the central response-selection stages of the shorter visual and the longer auditory task. Thus, dual-task costs should be reduced at post-test after hybrid practice primarily in the auditory task and less so in the visual task. To test these assumptions, we performed mixed measures ANOVAs on the RT and error data with the within-subject factors Testphase (pre-test vs. post-test), Trialtype (single-task trials vs. dual-task trials), Task (auditory, visual task) and the between-subject factor Group (hybrid group vs. single-task group). This ANOVA revealed a significant four-way interaction, *F*(1,14) = 8.528, *p* = 0.01, $\eta_{p}^{2}$ = 0.38, for the RT data, suggesting changes in dual-task costs that differed between the auditory and the visual task. Accordingly, we conducted subsequent ANOVAs with the factors Testphase, Trialtype, and Group separately for the auditory and the visual task to assess whether the different types of practice led to changes in dual-task costs in these different tasks.

**FIGURE 4:**
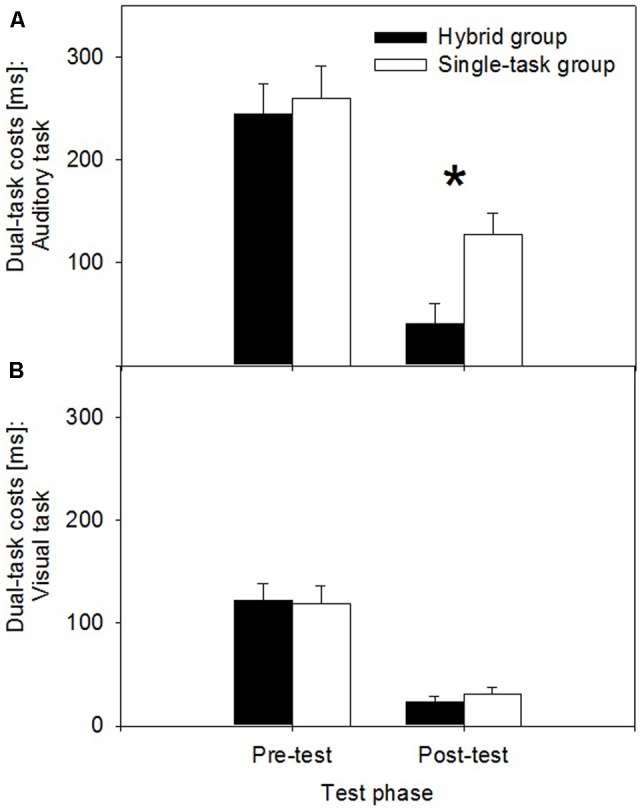
Dual-task costs in millisecond (ms; dual-task RTs minus single-task RTs) during pre-test and post-test for the single-task practice group (Single-task group) and the hybrid practice group (Hybrid group). **(A)** Auditory-task data; **(B)** Visual-task data. The asterisk denotes the significant difference.

The RT results of the *auditory task* point to the acquisition and transfer of improved task coordination skills after hybrid practice. In fact, we found a three-way interaction between Testphase, Trialtype, and Group, *F*(1,14) = 12.671, *p* < 0.01, $\eta_{p}^{2}$ = 0.48. As illustrated in **Figure 4A**, at post-test, dual-task costs were significantly reduced after hybrid practice (*M* = 40 ms) in contrast to single-task practice (*M* = 110 ms), *t*(14) < 2.135, *p* < 0.05. At pre-test, the difference between dual-task cost between the hybrid group and single-task group was not significant, *t*(14) = 1.305, *p* = 0.21. Thus, improved dual-task performance in the hybrid group at post-test cannot be explained by improved initial dual-task performance levels in this group relative to the single-task group. Furthermore, the improvement in dual-task performance is dual-task-specific, since it cannot be explained with differences in single-task RTs between groups, *t*(14) = 0.714, *p* = 49. The remaining effects and interactions in this RT analysis were as follows: Testphase, *F*(1,14) = 257.679, *p* < 0.001, $\eta_{p}^{2}$ = 0.95, Trialtype, *F*(1,14) = 150.129, *p* < 0.001, $\eta_{p}^{2}$ = 0.92, Group, *F*(1,14) = 0.046, *p* = 0.83, Testphase × Trialtype, *F*(1,14) = 183.536, *p* < 0.001, $\eta_{p}^{2}$ = 0.93, Testphase × Group, *F*(1,14) = 1.123, *p* = 0.31, Trialtype × Group, *F*(1,14) = 0.057, *p* = 0.82. The error analysis of the auditory task showed no three-way interaction of Testphase, Trialtype, and Group, *F*(1,14) = 0.518, *p* = 0.49. The remaining effects and interactions in this analysis were as follows: Testphase, *F*(1,14) = 1.070, *p* = 0.32, Trialtype, *F*(1,14) = 51.683, *p* < 0.001, $\eta_{p}^{2}$ = 0.79, Group, *F*(1,14) = 1.104, *p* = 0.31, Testphase × Trialtype, *F*(1,14) = 3.646, *p* = 0.08, Testphase × Group, *F*(1,14) = 0.014, *p* = 0.91, Trialtype × Group, *F*(1,14) = 2.649, *p* = 0.13 (**Table 2**).

In order to test whether, the advanced dual-task performance (i.e., decreased dual-task costs) in the auditory task after hybrid practice compared to single-task practice is based on only a few participants with mean values strongly deviating from those of the rest, we conducted a non-parametric test on the dual-task RT costs in the auditory task. This test includes the rank of each participant according to its dual-task costs in the auditory task and it ignores the absolute dual-task costs. A non-parametric Mann–Whitney *U* test showed a significant difference between the ranks of the hybrid group (mean rank = 5.88) and the single-task group (mean rank = 11.13), *p* < 0.05 (lower rank value indicates a lower amount of dual-task costs). This result shows that the present finding of reduced dual-task costs after hybrid practice is not the result of only a few outlier participants.

In the *visual task*, there was no advantage in the RT data and thus no evidence for the acquisition and transfer of improved task coordination skills after hybrid practice. This conclusion results from the finding of a non-significant three-way interaction of Testphase, Trialtype, and Group, *F*(1,14) = 0.874, *p* > 0.37 (**Figure 4B**). The remaining effects and interactions in this RT analysis were as follows: Testphase, *F*(1,14) = 80.229, *p* < 0.001, $\eta_{p}^{2}$ = 0.85, Trialtype, *F*(1,14) = 117.221, *p* < 0.001, $\eta_{p}^{2}$ = 0.89, Group, *F*(1,14) = 0.569, *p* = 0.46, Testphase × Trialtype, *F*(1,14) = 49.712, *p* < 0.001, $\eta_{p}^{2}$ = 0.78, Testphase × Group, *F*(1,14) = 0.909, *p* = 0.36, Trialtype × Group, *F*(1,14) = 0.774, *p* = 0.39. Analogous, the error analysis of the visual task also showed no interaction of Testphase, Trialtype, and Group, *F*(1,14) = 0.153, *p* = 0.70. The remaining effects and interactions in this analysis on error rates in the visual task were as follows: Testphase, *F*(1,14) = 0.050, *p* = 0.84, Trialtype, *F*(1,14) = 3.630, *p* = 0.08, Group, *F*(1,14) = 0.035, *p* = 0.85, Testphase × Trialtype, *F*(1,14) = 7.177, *p* < 0.05, $\eta_{p}^{2}$ = 0.34, Testphase × Group, *F*(1,14) = 1.610, *p* = 0.23, Trialtype × Group, *F*(1,14) = 2.119, *p* = 0.17 (**Table 2**). In sum, the present data pattern is consistent with the assumption of an acquisition of transferable task coordination skill and the assumption of a speed-up switching operation between tasks after hybrid practice.

### Follow-Up Analyses

At this point, critics may say that the transferable character of task coordination skills has not yet completely demonstrated. This is because the specific combination of component tasks in Session 16 was previously experienced in Sessions 1, 2, and 8. The dual-task performance advantage in the hybrid group may thus exclusively result from practice in these tree sessions and it might not result from learning processes during the other practice sessions and the related variations of the component tasks. To test this counter argumentation, we conducted a new group of 10 participants with single-task practice of eight sessions only. Importantly, the changes in the characteristics of the stimulus-response mappings in these eight sessions were identical to the changes in the hybrid group’s first eight sessions. In addition, this new single-task group had single-task practice in the first seven sessions (with the exception of a pre-test and its two mixed blocks in the beginning of Session 2) and performed single-task and mixed blocks in the final test Session 8. We compared the dual-task performance of this new single-task practice group with the dual-task performance of the hybrid training group in the 8th session. This comparison showed no main effect of Group and no interaction with Group for the analysis of the auditory-task RTs during pre-test and post-test under single-task and dual-task conditions, both *F*s(1,18) < 2.402, *p*s > 0.14, $\eta_{p}^{2}$s < 0.15. This finding is important because it shows equal dual-task performance of the hybrid and the *new* single-task practice group after eight training sessions, which include the sessions with identical stimulus-response characteristics of the component tasks as those in Session 16 of the hybrid group. The fact that we could not find a difference between the new single-task and hybrid group at Session 8, but a significant difference in dual-task performance between the (initial) single-task and the hybrid group at Session 16, suggests the latter dual-task advantage has occurred because of the additional training sessions between Sessions 9 and 16. However, the trained component tasks during these additional training sessions, i.e., Sessions 9–15, did differ from the component tasks in Session 16. Therefore, we can conclude that the observed hybrid-practice advantage after 16 sessions cannot be explained by the repetition of the component task situation in Session 16 with that from the Sessions 1, 2, and 8. Differently to that participants of the hybrid-practice task have acquired skills from the training with task situations that differed to those from the component tasks in Session 16 and the acquired skills have been transferred between task situations. The results showed further that this transfer requires more than eight sessions of practice in the current protocol of hybrid practice.

## Discussion

The present study investigated whether hybrid-practice-related task coordination skills are independent from the specific characteristics of the practiced component tasks and are thus transferable in a well-controllable practice and transfer context. In particular, transferable skills were shown in the data of the longer auditory task, but not for the data of the shorter visual task of the present task design when both component tasks were changed between practice and transfer. This data is in line with and extents the findings of Liepelt et al. (2011) as well as Strobach et al. (2015b) that provided evidence of skill transfer to dual tasks with only one changed task. These prior findings did not rule out that improved dual-task coordination skills may require constant features between practice and transfer, such as at least one non-changed component task. The present dual-task transfer test (Session 16) points to a hybrid-practice advantage with changed characteristics in two tasks. Furthermore, our data provide hints for the dose-dependency of transferable task coordination skills, since there is no hybrid-practice advantage after eight sessions when compared with the new single-task group. The hybrid-practice advantage in dual tasks emerged only after a doubling of the practice amount.

In general, our findings suggest that the automatization of the combined component tasks (e.g., Ahissar et al., 2001; Ruthruff et al., 2006; Maquestiaux et al., 2008; Strobach et al., 2013; Strobach and Schubert, 2017) is complemented by the acquisition of task coordination skills. Both mechanisms, i.e., task automatization and improvement of task coordination contribute to the practice-related optimization of dual-task processing (Hirst et al., 1980; Kramer et al., 1995). The present data suggest that task automatization has played a rather minor role in the present dual-task context since the component tasks of the post-test (i.e., Session 16) received a small dose of repetitions during prior sessions. Note that the component tasks of the post-test Session 16 were repeated only in Sessions 1, 2, and 8. In all other sessions, we changed the stimuli and stimulus-response mapping rules, which precluded a repeated learning of specific stimulus-response episodes, which, however, would be needed to enable task automatization (Ruthruff et al., 2006). Moreover, we found large transfer effects in the final Session 16, which was preceded by permanently changing component task situations especially from Session 8 and, partially, also during the Sessions 1–8.

One alternative explanation might be that hybrid practice serves to integrate two tasks more efficiently, to the point of combining them into one single ‘super task’ (Hazeltine et al., 2002; Ruthruff et al., 2006). According to this super task explanation, one might assume that two separate response-selections processes were performed at the beginning of practice, one response-selection in each component task, while the extensive hybrid practice might have led to the integration of two response-selection processes into one single selection process of a combined task. The processing of only one selection process, instead of two, would reduce dual-task RTs. In fact, the situation of separate practice of two tasks during single-task practice would have prevented integration of both selection processes and would thus prolong RTs in the dual-task situation. However, the integrated selection of two responses after hybrid practice should require that specific pairs of component tasks should be presented constantly throughout the training and that their specific combination should remain constant even in the post-test session; otherwise in case of permanently changing component tasks, including stimuli and stimulus-response rules, an integrated response-selection process could not emerge and could not transfer from one session to the next; the latter is prevented if the task rules have changed from session n-1 to n, which was precisely the case in the current hybrid training regimen (Hazeltine et al., 2002; Ruthruff et al., 2006). Because we found transfer of skills between changing task situations as a result of hybrid dual-task training, the observed practice-related improvement of dual-task performance cannot be explained by the assumption that both tasks were integrated into one super-task representation.

But how do task coordination skills acquired by participants improve dual-task performance exactly? As illustrated in **Figures 1A,B**, we assume that the present findings favor a shortened switching operation as a potential realization of improved skills of task coordination (Liepelt et al., 2011; Strobach et al., 2014). A shortened switching operation may be located at the end of the central response-selection stage in the shorter task and before the start of this stage in a longer task (Lien et al., 2003; Band and van Nes, 2006); thus, this shortened operation is particularly suited to explain the exclusive hybrid-practice advantage in the longer (auditory) task and the lacking advantage in the shorter (visual) task. Such a location of the switching operation would be in accordance with the assumption that training may lead to an optimized bottleneck processing being it a structural or strategic in nature (Pashler, 1994; Meyer and Kieras, 1997). A shortened switching operation may relate to a more efficient release (for example, by inhibition) of task information from the shorter task (that turns to an irrelevant task after the switch in a current trial) as well as the activation and instantiation of the response mapping rules of the longer task (De Jong, 1995). Due to its particular locus at the end of central processing in task 1, the shortening of a switching operation after hybrid practice would influence dual-task RTs in the longer auditory task, whereas there should be no (or only a minimal) effect on the shorter visual task of the present dual-task situation. These assumptions may explain the observed processing advantage in the current dual-task situation after hybrid practice.

Additionally, we assume that the proposed mechanism is generalizable in the following way: the shortening of a switching operation after hybrid practice would also influence dual-task RTs in any longer task (i.e., the task with the second response), while there should be no (or only a minimal) effect on any shorter task (i.e., the task with a first response); this generalization is based on the assumption that the order of motor responses is equivalent to the order of the tasks’ response-selection stages (Ruthruff et al., 2006). In that case, a hybrid practice effect might lead especially to an earlier start of a task 2 response after the switch and the occurrence of this dual-task training effect should be independent on the specific stimulus and response-selection characteristics of task 2 and task 1 as long as the order of a shorter and a longer task is preserved throughout the training (Strobach et al., 2014). While the current experiment provided evidence for this assumption for the combination of a certain order of a shorter visual motor and an auditory verbal task, other studies may test whether other task combinations would allow for the occurrence of a hybrid dual-task practice advantage located at the longer task (or task 2) of a dual-task situation.

In sum, we demonstrated that task coordination skills improving dual-task performance with practice, are (1) acquired in dual-task situations, (2) transferable, and (3) dose-dependent. Future studies may specify this type of skill acquisition (Taatgen, 2013) and locate its impact in the dual-task processing architecture (Meyer and Kieras, 1997; Schubert, 2008; Strobach et al., 2014).

## Ethics Statement

Approval by the local ethics committee was obtained before commencement of the study, which was conducted in strict accordance with the local ethics policies of the Humboldt University Berlin, Germany.

## Author Contributions

ToS, RL, SK, and TiS were involved in every step of this research project.

## Conflict of Interest Statement

The authors declare that the research was conducted in the absence of any commercial or financial relationships that could be construed as a potential conflict of interest.
